# Delineation of VEGF-regulated genes and functions in the cervix of pregnant rodents by DNA microarray analysis

**DOI:** 10.1186/1477-7827-6-64

**Published:** 2008-12-16

**Authors:** Chishimba N Mowa, Tianbo Li, Subrina Jesmin, Hans G Folkesson, Sharon E Usip, Raymond E Papka, Guichuan Hou

**Affiliations:** 1 Appalachian State University, Department of Biology, Rankin Science North Building N219, 572 River Street, Boone, NC 28608, USA; 2 Northeastern Ohio Universities Colleges of Medicine and Pharmacy, Departments of Integrative Medical Sciences, 4209 St. Rt. 44, PO Box 95, Rootstown, Ohio 44272, USA; 3 Department of Gene Diagnostics and Therapeutics, Research Institute, International Medical Center of Japan, Tokyo, 1-21-1 Toyama, Shinjuku-ku, 162-8655, Tokyo, Japan; 4 Northeastern Ohio Universities Colleges of Medicine and Pharmacy, Departments of Neurobiology and Anatomy, 4209 St. Rt. 44, PO Box 95, Rootstown, Ohio 44272, USA

## Abstract

**Background:**

VEGF-regulated genes in the cervices of pregnant and non-pregnant rodents (rats and mice) were delineated by DNA microarray and Real Time PCR, after locally altering levels of or action of VEGF using VEGF agents, namely siRNA, VEGF receptor antagonist and mouse VEGF recombinant protein.

**Methods:**

Tissues were analyzed by genome-wide DNA microarray analysis, Real-time and gel-based PCR, and SEM, to decipher VEGF function during cervical remodeling. Data were analyzed by EASE score (microarray) and ANOVA (Real Time PCR) followed by Scheffe's *F*-test for multiple comparisons.

**Results:**

Of the 30,000 genes analyzed, about 4,200 genes were altered in expression by VEGF, i.e., expression of about 2,400 and 1,700 genes were down- and up-regulated, respectively. Based on EASE score, i.e., grouping of genes according to their biological process, cell component and molecular functions, a number of vascular- and non-vascular-related processes were found to be regulated by VEGF in the cervix, including immune response (including inflammatory), cell proliferation, protein kinase activity, and cell adhesion molecule activity. Of interest, mRNA levels of a select group of genes, known to or with potential to influence cervical remodeling were altered. For example, real time PCR analysis showed that levels of VCAM-1, a key molecule in leukocyte recruitment, endothelial adhesion, and subsequent trans-endothelial migration, were elevated about 10 folds by VEGF. Further, VEGF agents also altered mRNA levels of decorin, which is involved in cervical collagen fibrillogenesis, and expression of eNO, PLC and PKC mRNA, critical downstream mediators of VEGF. Of note, we show that VEGF may regulate cervical epithelial proliferation, as revealed by SEM.

**Conclusion:**

These data are important in that they shed new insights in VEGF's possible roles and mechanisms in cervical events near-term, including cervical remodeling.

## Background

Cervical remodeling is considered a chronic inflammatory-like process regulated by numerous factors, and its dysfunction can potentially lead to birth-related complications [1-4]. Because the vasculature plays a crucial role in inflammatory reactions, we have previously hypothesized that factors that regulate the cervical vasculature are likely to play a critical role in cervical remodeling, notably VEGF and its associated molecules, such as nitric oxide. For instance, local microvascular alterations during cervical remodeling may be essential for delivery of cells and factors to the connective tissues for remodeling. In turn, vascular-derived factors, such as leukocytes, play a critical role in cervical remodeling by invading cervical tissue and releasing catabolic enzymes and cytokines [5]. Thus, recruitment or mobilization of leukocytes into the cervical connective tissue may require structural changes to the vasculature, and this process may be regulated, directly and/or indirectly, by several factors.

VEGF is a member of a family of closely related growth factors that include VEGF-A, -B, -C, -D, -E and placenta growth factor (PIGF) [6]. VEGF-A has well-established biological effects and exists as several splice variants [6]. Biological effects of VEGF are largely mediated by two receptors: KDR (kinase domain region) and Flt-1 (fms-like tyrosine kinase-1) [7,8]. The role of VEGF in female reproductive biology is best known in the ovarian and uterine events. VEGF is essential for a variety of ovarian and uterine endometrial functions by mediating cyclical growth of blood vessels. For instance, treatment with a VEGF inhibitor (mFlt- [1-3]-IgG) virtually blocks *corpus luteum *angiogenesis and maturation of endometrium [9]. VEGF signaling pathways for microvascular regulation have been extensively studied to date, mostly in human umbilical vein endothelial cells [HUVECs]. In spite of this, very little is known about VEGF function in the cervix in general and cervical remodeling in particular. We recently reported that only VEGF variants 120 and 164 exist in the rat cervix [10]. In general, VEGF 164 is the most abundant and best characterized of all VEGF variants in the body. We also demonstrated that there exist two VEGF receptors in the cervix of pregnant rats, namely KDR and Flt-1, and that VEGF, its receptors, and some of its key signaling molecules are altered in the cervix during pregnancy [10].

Although the mechanisms mediating specific vascular effects of VEGF are beginning to be unraveled, they are not fully elucidated and vary between vascular beds. A global or genome-wide view of VEGF-related genes in the "ripening" cervix and knowledge of the specific VEGF/VEGF receptor pathway mediating their cellular effects, is essential for obtaining a comprehensive evaluation of the processes (vascular and non-vascular) regulated by VEGF. In this study, we alter VEGF action by either over-expressing, down regulating or blocking VEGF action in the cervix of non-pregnant and pregnant rodents (rat and mice) using recombinant VEGF-protein, -siRNA generating pDNA or -receptor antagonist (PTK787), respectively. Tissues were analyzed using DNA microarray, gel-based PCR, Real-Time PCR, SEM, and histology.

## Methods

### Animals and treatment with VEGF agents

a) *Timed-pregnant Sprague Dawley rats *[[17-20] gestation day (GD); SASCO strain from Charles Rivers] were divided into four groups (n = 5), based on treatment: a) VEGF siRNA-generating pDNA (group 1; 40 μg/rat on alternate days), b) VEGF inhibitor (PTK 787/ZK22584; generously provided by Novartis Pharma AG, Basel, Switzerland) (group 2; 1, 2.5, 5 mg/rat/on alternate days), c) vehicle only (VEGF inhibitor control; group 3), and d) vehicle with plasmid without VEGF siRNA insert (VEGF siRNA control; group 4). The chemicals/nucleic acids were pre-dissolved in 100 μl lipofectamine and mixed in 100 μl saturated (25%) Pluronic F-127 (Sigma, St Louis) on ice. The solution was then administered to the cervix via the vagina using a 1-mL syringe on alternate days from GD17 to GD19. The animals were euthanized by a lethal injection of sodium pentobarbital (150 mg/kg bw), i.p., 12–24 hours later, on GD 20. Cervices were harvested, processed and evaluated for changes in levels of VEGF mRNA and whole genome gene expression using DNA microarray data, as described below.

b) *Pregnant mice and ovariectomized non-pregnant rats and mice *treated with either mouse recombinant VEGF protein (10 ng/mouse recombinant VEGF; Calbiochem, La Jolla, CA, once daily from GD13–17, intra-vaginally), VEGF receptor antagonist (as described earlier, but treated daily), or vehicle only, were utilized to confirm the DNA microarray data above using real-time PCR, and for morphological studies (SEM), described below. The animals were euthanized by a lethal injection, as described earlier. Cervices were harvested, processed and evaluated for changes in levels of select genes (fold change > 2), and SEM.

### Generation and validation of VEGF siRNA-generating pDNA

#### Generation

We designed three pairs of VEGF siRNA oligos, annealed and ligated them with a *Bam*HI/*Hind*III restricted pSilencer-3.0-H1 vector, separately. The recombined vectors were transferred into *E. coli *DH5α, grown on ampicillin (100μ/ml) LB dishes at 37°C over night. The clone for each pDNA was obtained and confirmed by sequencing.

#### Validation

The VEGF gene silencing efficiency by the siRNA-generating pDNA was tested *in vitro*. Ten μg (in 250 μl water) of each siRNA-generating pDNA was mixed with 10 μl Lipofectamine 2000 (Eugene, Oregon) and used for rat heart fibroblast primary cell transfection. The pDNA with the best silencing efficiency was selected and used in the rat cervix with either pluronic gel or Lipofectamine 2000, to test its efficiency to down-regulate local cervical VEGF *in vivo*.

### Gene expression analysis

#### Overview

Changes in mRNAs levels of various genes in the cervices of rats treated with VEGF agents were quantified using gel-based PCR, DNA microarray, and real time PCR. Gel-based PCR was also utilized to determine VEGF gene silencing efficiency by the pairs of VEGF siRNA oligos in the rat heart fibroblasts *in vitro*. DNA microarray data were verified by real-time PCR.

#### Tissue processing and messenger RNA isolation

The animals were euthanized and transcardially perfused with normal saline. The cervices were removed and stored at -80°C until processing. Total RNA was isolated from cervices of individual animals using RNeasy Mini Kit (Qiagen, Valencia, CA). The amount and purity of total RNA for each sample was estimated by spectrophotometric analysis at A260 and A280. The quality of RNA was determined by agarose gel electrophoresis following SYBR green™ (Invitrogen) staining. Aliquots of total RNA were diluted in diethylpyrocarbonated (DEPC)-treated water and stored at -80°C. The RNA quality and quantity for DNA microarray analysis was determined, as described under the DNA microarray section.

#### a) Gel-based PCR analysis: *RNA preparation, RT-PCR and Gel-based PCR*

Gel-based PCR was employed to determine VEGF gene silencing efficiency by the pairs of VEGF siRNA oligos in the rat heart fibroblasts *in vitro *and the cervices of rats treated with VEGF siRNA and control, as described earlier. Total RNA from these two tissue-types was reverse transcribed and amplified in an Eppendorf Master Cycler, using reagents from Gene AMP Gold RNA PCR Kit (Perkin Elmer BioSystems, Foster City, CA). The RNA was used in RT-PCR to evaluate the total VEGF-A gene using as primers 5'-GTACCTCCACCATGCCAAGT-3' (sense) and 5'-GCATTAGGGGCACACAGGAC-3' to generate a PCR product of 194 bp [11]. Levels of VEGF genes were normalized to the geometric mean of the internal control gene, glyceraldehyde-3-phosphate dehydrogenase (GAPDH).

#### b) DNA microarray: *Determination of RNA integrity and microarray statistical analysis*

Integrity of total RNA was evaluated using capillary electrophoresis (Bioanalyzer 2100, Agilent) and quantified using a Nanodrop 1000 (Nanodrop, Wilmington, DE). Following confirmation of RNA quality, Ovation™ Biotin RNA Amplification and Labeling System (NuGen Technologies, Inc., San Carlos, CA) was used to prepare amplified, biotin-labeled cDNA from total RNA following manufacturer's instructions. Briefly, first strand cDNA was synthesized from 25 ng of total RNA using a unique first strand DNA/RNA chimeric primer and reverse transcriptase. Following double strand cDNA generation, amplification of cDNA was achieved by utilizing an isothermal DNA amplification process that involves repeated SPIA™ DNA/RNA primer binding, DNA duplication, strand displacement and RNA cleavage. The amplified SPIA™ cDNA was purified and subjected to a two-step fragmentation and labeling process. The fragmented/biotinylated cDNA content was measured in a ND-1000 spectrophotometer and the quality was analyzed on an RNA 6000 Nano LabChip (Agilent) using Agilent Bioanalyzer 2100. For each array, 2.2 μg of cDNA was hybridized onto Rat Genome 230 2.0 GeneChips^® ^(Affymetrix Inc., USA), containing more than 31,000 probe sets, analyzing over 30,000 transcripts and variants from over 28,000 well-substantiated rat genes. Sequences used in the design of the GeneChip Rat Genome 230 2.0 Array were selected from GenBank^®^, dbEST, and RefSeq. The sequence clusters were created from the UniGene database (Build 99, June 2002) and then refined by analysis and comparison with the publicly available draft assembly of the rat genome from the Baylor College of Medicine Human Genome Sequencing Center (June 2002). Hybridization was allowed to continue for 16 hours at 45°C, followed by washing and staining of microarrays in a Fluidics Station 450 (Affymetrix Inc., USA). GeneChips were scanned in a GeneChip Scanner 3000 (Affymetrix Inc., USA) and CEL files were generated from DAT files using GeneChip^® ^Operating Software (GCOS) software (Affymetrix Inc., USA). The probe set signals were generated using the RMA algorithm in ArrayAssist 3.4 (Stratagene) and were used to determine differential gene expression by pair-wise comparisons. The genes that were altered by two-fold either way were sorted and used for further interpretation of the microarray data, and were also analyzed using the *Expression Analysis Systematic Explorer *(EASE), a software application that facilitates biological interpretation of gene lists derived from microarray, proteomic, and SAGE experiments . EASE helps identify biological themes that are over-represented in array results. Finally, data were analyzed using the cluster and scatter plots (data not shown).

#### c) Real-time PCR. Real-time PCR procedure and statistical analysis

Real-time PCR was used to verify DNA microarray data. Total RNA was DNase I treated, reverse transcribed, and amplified as described above. The cDNA was then used to evaluate the relative expression of select genes with fold change of equal or greater than 2, including VEGF-A, eNOS, decorin, PLCγ, PKC, VCAM-1, and oxytocin using specific TaqMan Gene Expression Assays (Applied Biosystems), which are pre-designed and pre-optimized gene-specific probe sets. DNA amplification was performed using the Applied Biosystems (ABI 7300 HT) Real-Time PCR machine with the GeneAmp 7300 HT Sequence detection system software (Perkin-Elmer Corp.). The PCR reactions were set up in wells of a 96-well plate in a volume of 50 μl per well. The reaction components were: 2 μl of synthesized cDNA, 25 μl of 2 × TaqMan Universal PCR Mastermix, 2.5 μl of 20 × Assays-on-demand™ Gene Mix (e.g., VEGF), and 20.5 μl of RNase-free water. The program was set as follows: an initial step at 95°C for 10 min, and then 40 cycles of 95°C for 15 s, and 60°C for 60 s. The relative amount was calculated from the threshold cycles with the instrument's software (SDS 2.0), according to the manufacturer's instructions. Relative expression levels of the target genes were normalized to the geometric mean of the internal control gene, GAPDH. Data were analyzed using Student's *t *test and ANOVA, followed by Scheffe's *F*-test for multiple comparisons. *P*-values of < 0.05 were considered to be statistically significant.

### Morphological studies

SEM studies were undertaken to visualize the effects of VEGF agents on cervical morphology. Tissues were fixed in 2.5% glutaraldehyde in 0.1 M PBS immediately after sampling, and then washed in the buffer (0.1 M PBS). After the washes, tissues were dehydrated in a graded ethanol series, and dried with a Polaron critical point drying apparatus (Polaron Instruments Inc., Doylestown, PA). All dried samples were mounted on aluminum stubs, sputter coated with gold, and imaged with a Quanta 200 SEM (FEI Company, Hillsboro, OR) at 20 kV.

## Results

### VEGF gene silencing efficiency by siRNA-generating pDNA

#### a) Rat heart fibroblast primary cell

**Figure 1:**
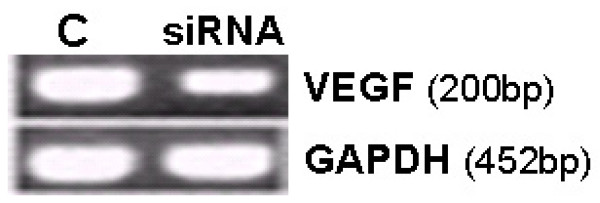
**VEGF siRNA-generating pDNA down regulates VEGF mRNA levels in rat heart fibroblasts**. The VEGF gene silencing efficiency by the three siRNA-generating pDNAs were tested *in vitro *using rat heart fibroblast primary cell transfection. VEGF siRNA-generating pDNA was found to down-regulate VEGF in cultured rat heart fibroblasts compared to control. The pDNA with the best silencing efficiency was selected and used in the rat cervix to test its efficiency to down-regulate local cervical VEGF *in vivo *(see Figure 2). n = 5, (*p *< 0.05).

The three different pairs of VEGF siRNA-generating pDNA transfected to rat heart fibroblast primary cell had different silencing efficiency for VEGF. The pair with the best efficiency was used in subsequent experiments (Figure 1).

#### b) Cervices of pregnant rats

**Figure 2:**
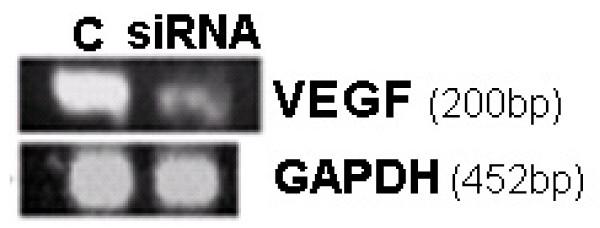
**VEGF siRNA-generating pDNA down regulates VEGF mRNA levels in rat cervix**. The VEGF gene silencing efficiency of the selected siRNA-generating pDNA was tested *in vivo *in the cervix of pregnant rat from GD17–20. VEGF siRNA-generating pDNA was found to down-regulate VEGF compared to control. n = 5, (*p *< 0.05).

VEGF siRNA-generating pDNA delivered to the cervix on alternate days from GD17 to GD19 down-regulated about half of VEGF mRNA by GD20, as revealed by gel-based PCR data (Figure 2). Grossly, the cervices of rats treated with VEGF siRNA appeared paler, compared to control (data not shown). We interpreted this to mean reduced vascular formation due to reduced VEGF levels. This interpretation was also supported by the DNA microarray data (See Table 1 and additional file 1 for the original data used to perform this analysis).

**Table 1:** VEGF inhibitor (PTK787) alters various biological themes in cervix of pregnant rat.

**Gene Category**	**Ease score**
Steroid Biosynthesis	+6.11
Steroid Metabolism	+4.97
Protein Kinase Activity	~+4
Cell Proliferation	-8.11
Cell Motility	-7.66
Circulation	-3.16
Tissue Remodeling	-3.16
Calcium Channel	-2.5
Immune Response	-4.70
Heat Shock Protein Activity	-8.67
Cell Adhesion Molecule Activity	-1.96

The biological processes, cellular components or molecular functions altered when VEGF action is blocked (VEGF inhibitor) during cervical remodeling (GD20), as revealed by DNA microarray analysis. n = 5.

### Genome-wide delineation of VEGF-related genes by DNA microarray

#### DNA microarray analysis

As described earlier, gene expression profile of 30,000 rat genes in the cervix of pregnant rats were analyzed following intra vaginal treatment of rats during late gestation with VEGF agents (siRNA and inhibitor).

#### VEGF agents

Out of a total of 30,000 genes, about 4,200 genes had altered gene expression at GD20, after intra vaginal administration of the VEGF inhibitor. Of these, gene expression of about 1,700 genes were up regulated (*p *< 0.05), while the rest, slightly over 2400, were down-regulated (*p *< 0.05). These genes are annotated in the database of NCBI's Reference Sequence project and are also found on the annotation list of Code link. Although there was a match between genes altered by VEGF siRNA and blocker, the impact of VEGF siRNA, with an efficiency of about 50%, on gene expression was significantly less pronounced compared to receptor antagonist. Only expression of about 300 genes were altered by VEGF siRNA, of which about 50 were up regulated and about 220 were down-regulated. Thus, henceforth, we will only focus on data from VEGF receptor antagonist-treated animals.

#### Biological classification

We analyzed the data based on the EASE score, a robust analytical tool that clusters altered gene expressions based on cell component and molecular biological functions. It was interesting to note that, based on this score, the "hallmark" biological processes of VEGF, and most processes that may be relevant to cervical remodeling, were connected and or regulated by VEGF in the cervix. This included cell proliferation and motility, circulation, immune and inflammatory responses, protein kinase activity, calcium channel activity, tissue remodeling, heat shock protein activity, and cell adhesion molecule activity (See Table 1). Other processes, not "mainstream" to VEGF-related processes, but known to play a role in cervical remodeling includes, steroid synthesis and metabolism (See Table 1) and collagen metabolism.

Select groups of genes altered by VEGF agents include, growth factors (e.g., EGF, FGF, IGF binding protein 1, PGF, PDGF receptor, and TGFβ-1), inflammation-associated factors (e.g., chemokines, LPS binding proteins, interferons, interleukins, and TNF), cervical matrix factors (e.g., proteoglycans, hyaluronan acid, heparan sulfate, and pro-collagen and collagen molecules), signaling molecules (e.g., PLCγ, PKC, AC 5, MAPKK 6, guanine nucleotide binding protein 13γ), neuronal factors (e.g., GABA A receptor, galanin receptor 2, and α2a-adrenergic receptor), vascular factors (e.g., VEGF C, endothelin receptor type A, angiotensin II receptor, type 1, and angiopoietin 4), and factors involved in WBC migration (e.g., integrins and a variety of cell adhesion molecules) (See additional file 1 for the original data used to perform this analysis).

#### Real-time PCR analysis

**Figure 3:**
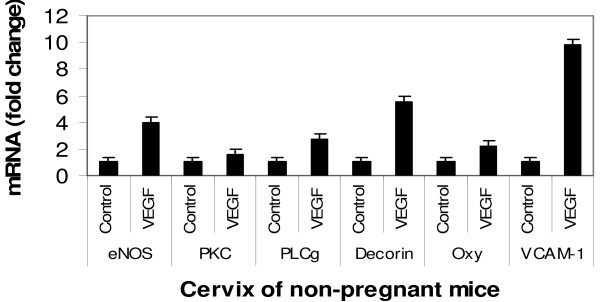
**Mouse recombinant VEGF protein 164 up regulates gene expression of some genes in mouse cervix**. Levels of eNOS, PKC, PLCγ, Decorin, Oxytocin and VCAM-1 mRNAs in the cervix of non-pregnant ovariectomized mice were up regulated by recombinant VEGF protein164 (10 ng/mice × 3 times/day for 3 days), as revealed by Real time PCR. n = 3, (*p *< 0.05).

Real-time PCR was used to verify DNA microarray data for 6 genes, namely VCAM-1, decorin, PLCγ, PKC, oxytocin, and eNOS [See Figure 3 and additional file 1 for the original data used to perform this analysis]. Data generated from Real-time PCR analysis were found to be consistent with the results from the DNA microarray.

#### SEM

**Figure 4:**
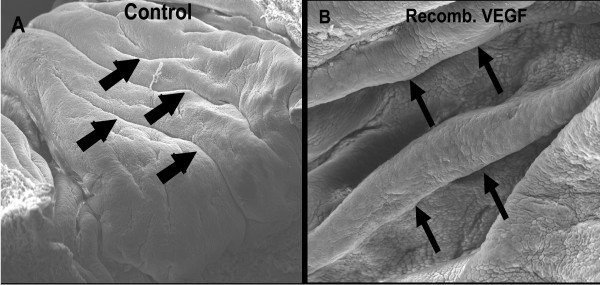
**Mouse recombinant VEGF protein 164 induces epithelial growth in mouse cervix**. The effects of VEGF protein (mice recombinant VEGF164 protein, 10 ng/mouse × 3 times/day for 3 days, intravaginally) on cervical epithelial proliferation growth of non-pregnant ovexed mice, as revealed by SEM (A. Control, B, VEGF protein). Note pronounced involutions in treated (thin arrows) compared to shallow folds in control (thick arrows). Mag × 3,700. n = 3.

SEM data demonstrated a pronounced increase in the involutions of cervical epithelial sheet in VEGF protein-treated mice compared to control (non-pregnant ovariectomized) (Figure 4).

## Discussion

Dysfunctional cervices are among the most important etiologies of obstetrical complications, such as preterm and protracted labor. Numerous studies have investigated the roles of hormones, cytokines, prostaglandins, relaxin, and leukocytes in cervical remodeling. However, the lack of a comprehensive understanding of the exact factors triggering cervical remodeling and effective treatments emphasizes the need to explore and identify new targets for therapeutic interventions, such as vascular factors. To date, virtually none have studied the role of cervical microvasculature in cervical remodeling and how it may impact parturition. The present study was designed to identify signature genes that are commonly altered in cervices of non-pregnant and pregnant rodents (mice and rats) and the impact of altered VEGF action (blockade and over-expression) on gene expression and morphology of the cervix. The findings in these studies are novel and important in that they address basic informational gaps and demonstrate, for the first time, the potential role of VEGF in cervical remodeling and factors involved in VEGF action. However, the limitations of the present data are that they only show alterations in mRNA levels and not protein.

We previously demonstrated an association between VEGF and its key signaling components, and based on these data postulated that since vascular-derived factors play important roles in cervical remodeling in general, local microvascular alterations are essential for delivery of these factors to cervical connective tissues for remodeling to occur. Such vascular alterations include increased vascular leakage; vasodilation, and vascular density and expansion (angiogenesis); accompanied by alterations in the synthesis of angiogenic factors [10,12]. Collectively, these vascular changes increase the overall traffic of intravascular factors, such as serum factors, fluids, nutrients, and effector cells, and likely, in turn, enhance epithelial cellular growth and proliferation and, thus, impact cervical remodeling. These structural and molecular alterations in the vasculature are re-set to baseline levels immediately after birth [10]. This postulation is consistent with data from a later study that showed that VEGF alters the biomechanical properties of the cervix by diminishing tensile strength [13].

To fully understand the functional role of VEGF, its pathways regulating cervical remodeling and microvascular events, it is critical to map and identify "signature" genes down-stream of or regulated by VEGF. This knowledge would "*fast-track" *our efforts and will enable us to focus on a small select number of VEGF- regulated genes. Moreover, it will expand our understanding of the relationship between "*signature*" genes and the well-known regulators of cervical remodeling, such as prostaglandins, IL-1, and platelet growth factor that interestingly, are also known to regulate VEGF in other tissues of the body [14-16]. It is interesting to note that, based on the grouping of genes according to their biological process, cell component, and molecular functions using the EASE score, the "hallmark" biological processes of VEGF, and most processes which are relevant to cervical remodeling, are present and regulated by VEGF in the cervix. These processes include, protein kinase activity, cell proliferation and motility, circulation, ion channel activity, heat shock protein activity, immune response, and cell adhesion molecule activity. Other processes, not "mainstream" VEGF-related processes, include regulation of tissue remodeling, steroid synthesis, and metabolism.

Signaling mechanisms that underlie VEGF-induced vascular permeability appear to be context-and tissue-dependent and are, in general, unclear [17]. For instance, VEGF-induced formation of fenestrae is mediated by NO and prostacyclin (PGI_2_), in some tissues [18,19], and inhibitors of synthetic enzymes for these molecules (e.g., the eNOS inhibitor L-NAME [a non-selective NOS inhibitor that also inhibits inducible- and neuronal-NOS isoforms]) block VEGF-induced permeability changes *in vivo *[20]. In contrast, VEGF-induced rat uterine venous hyperpermeability involves calcium and phospholipase C-γ1 (PLCγ1), but not NO [21]. In the uterus, the best studied signaling transduction believed to mediate VEGF-induced vascular leakage, is mediated by PLC-γ1/DAG/IP3 pathway, which leads to increased calcium influx and induction of the PKC pathway [21]. This signaling pathway appears to be the most common pathway [21-25]. Interestingly, here we show that VEGF agents (recombinant protein and blocker) in the cervix alter mRNAs of eNOS, PKC, and PLC-γ¹, using DNA microarray, and verified by real time PCR. Thus, based on these data, VEGF likely regulates vascular leakage during cervical remodeling. Also, of interest, the present DNA microarray data show that several groups of cell adhesion molecules (ICAM, integrin, VCAM-1, melanoma CAM, Cerebral endothelial CAM) that play an essential role in WBC migration from blood vessels to tissues are robustly regulated by VEGF agents. Real time PCR data verified microarray data using VCAM-1. However, at this point the exact pathways that regulate cervical vascular permeability are not yet clear.

Other factors of interest that were altered by VEGF agents include inflammatory, matrix, neuronal, vascular, and growth factors. We have previously shown that sensory neuronal factors influence cervical events, and that bilateral neurectomy of the pelvic nerves diminished VEGF protein levels in the cervix of pregnant rats [12]. Here, we show that VEGF blocker also alters levels of neuronal factors in the cervix, suggesting a bidirectional or feedback regulatory mechanism. It is also interesting to observe that VEGF agents alter mRNA levels of notable inflammatory factors, such as TNFα, LPS binding protein, and IL-1, which is consistent with the proposition that cervical remodeling is essentially a physiological inflammatory response [4]. These data are also consistent with our recent data showing that local induction of inflammation in the cervix by LPS induces increased levels of VEGF mRNA [26]. Equally interesting is that fact that VEGF also alters mRNA levels of key matrix factors known to be involved in cervical remodeling, including decorin, collagen, biglycan, hyaluronic acid, and heparin sulfate. Collectively, these data, taken together with the biomechanical data by Dussably *et al*., [13], strongly suggests a role for VEGF in cervical remodeling.

Cervical remodeling is associated with increased epithelial proliferation and edema [27,28]. In mice, cervical edema is noticeable as early as GD12 and plateaus between GD15–19, and, immediately thereafter, reaching baseline levels by postpartum day 1 [29]. The exact function of edema and the mechanisms regulating its formation are not fully known. However, a number of factors have been implicated, including, prostaglandins, sex steroid hormones, relaxin, and aquaporins (subtypes 3, 4, 5 and 8) [30]. Furthermore, cervical epithelial cells, acting via paracellular apical tight junction protein complex, are known to maintain fluid balance (e.g., claudin 1 and 2) [31]. One of the best studied factors implicated in edema formation is hyaluronan, because of its high affinity for water molecules, and because its levels increase dramatically near term [32]. It has been proposed that hyaluronan during cervical remodeling could modify tissue architecture by increasing its volume and recruiting inflammatory cells [32]. However, in mice, levels of hyaluronan only significantly increase a few hours before labor, whereas edema increases much earlier [30]. Here, we show by microarray analysis that VEGF receptor antagonist down regulates mRNA levels of hyaluronan. Thus, we suggest VEGF as an additional candidate regulator of cervical edema that may work together with or independent of hyaluronan for the following reasons: a) vascular permeability is the major contributor of tissue hydration and VEGF, which was originally identified as an inducer of vascular permeability factor from a guinea pig cell line [33], is the most potent permeability factor known in the body (50,000 times more potent than histamine)[34,35]; b) VEGF-induced vascular permeability is a major factor underlying significant tissue hydration in multiple tissues [36]; c) VEGF has been demonstrated to induce vascular permeability via altering cellular junctions of endothelial cells, e.g., creating fenestrae through which intravascular solutes and fluids pass into the tissue [36,37]; d) VEGF and its signaling molecules are present in the remodeling cervix and have a temporal relationship with edema; e) In the cervix, VEGF agents induce factors that are known to mediate its vascular permeability effects, such as NO and PKC; c) and finally, vascular leakage occurs during cervical remodeling. Thus, here we postulate a working model that suggests that during cervical remodeling, VEGF-induced vascular leakage lead to protein extravasation, which in turn, due to their strong hydrophilic properties (albumin and fibrin), attract serum into the cervical tissue, and thus leading to tissue hydration, cervical epithelial proliferation [38]. Further studies should validate this model and the proposed effects of VEGF on cervical edema, as is seen in most body tissue-types.

Cervical cellular activity and proliferation are particularly pronounced in endocervical epithelial cells, i.e., epithelial cells proliferate during pregnancy to occupy ~50% of the entire cervix [30]. Cervical epithelial cells are now believed to be involved in multiple and important functions, during cervical remodeling, including: 1) maintaining fluid balance via synthesis of hydrophilic hyaluronan, glycosaminoglycan, and aquaporins, 2) proliferation and differentiation, 3) regulation of paracellular transport of solutes via tight junctions, 4) providing a protective barrier against invading micro-organisms, and mediating inflammatory and adaptive immune responses, 5) acting as an "endocrine" gland by synthesizing prostaglandins, chemokines and cytokines (e.g., interleukin-8), and steroid hormones [39-42,30]. Thus, it is likely, as some investigators suggest, that cervical epithelial cells play the central role in cervical remodeling. The best studied regulators of cervical epithelial cell proliferation are relaxin and sex steroid hormones [43]. However, the growth and proliferation of these cells could also be regulated by other factors, either in concert or independent of relaxin and sex steroid hormones. For instance, the classical uterine response to 17β-estradiol, namely increased epithelial cell growth and proliferation, is mediated by VEGF via increased vascular permeability and edema [44], and is the case in other multiple tissue types [45-47]. Thus, it seems that this phenomenal proliferation is wide spread in the body. VEGF is known to induce epithelial growth and proliferation during implantation, in fetal lung, prostate, and also induces astroglial cell division in the brain [45-47] via two mechanisms: 1) VEGF may stimulate endothelial cells to secrete growth factors (e.g., FGF, IGF, and PDGF) that, in turn, stimulate proliferation of neighboring epithelial cells [48], or 2) VEGF may induce vascular permeability that leads to increase in infiltration of local tissue and induction of epithelial growth by serum factors, which are a rich source of growth factors [49]. This later mechanism is supported by well established facts that cells are functionally more active when cultured *in vitro *in the presence of serum, and solid epithelial tumor cells lacking adequate access to a blood supply will grow only until passive diffusion can no longer provide adequate nutrients [50,51,49]. Both mechanisms may be operational in the cervix, and may, in part, account for the phenomenal epithelial proliferation during cervical remodeling, as revealed by our SEM data. Ongoing studies in our lab are quantifying the effects of VEGF on epithelial proliferation.

## Conclusion

Here, we show that VEGF regulates levels of key factors involved in a range of biological processes that lead to cervical remodeling. We also show that VEGF induce cervical epithelial growth. These data are novel and important in that they shed new insights in VEGF's possible roles and possible underlying mechanisms during cervical remodeling.

## Competing interests

The authors declare that they have no competing interests.

## Authors' contributions

CNM initiated and designed the project, treated and harvested tissues from animals, data analysis and prepared the manuscript. TL and HGH performed and analyzed the siRNA work and performed gel-based PCR on siRNA-treated rat heart fibroblast cells. SJ helped design the original project, data analysis and manuscript preparation. SEU performed part of the gel-based PCR and helped with treating and harvesting rat tissues. REP provided initial funds and guidance in the initial stage of the project. GH performed SEM and analyzed the data.

## Supplementary Material

Additional file 1 **Table 2**: Selected list of genes altered by VEGF inhibitor (PTK787) in cervix of pregnant rat. Click here for file
