# Supplementary material for: Delineation of VEGF-regulated genes and functions in the cervix of pregnant rodents by DNA microarray analysis
Source: Reprod Biol Endocrinol. 2008 Dec 16;6:64. doi: 10.1186/1477-7827-6-64 (PMC2628666; doi:10.1186/1477-7827-6-64)
Supplement: Additional file 1 — Table 2: Selected list of genes altered by VEGF inhibitor (PTK787) in cervix of pregnant rat. [file 1477-7827-6-64-S1.doc]

**Table 2.** *Selected list of genes altered by VEGF inhibitor (PTK787) in cervix* *of pregnant rat* The changes in expression (selected) of pregnancy-dependent genes (1=GD10 vs. GD20) and VEGF-regulated genes (VEGF blocker [2] Vs GD20) when VEGF action is blocked in the cervix during pregnancy (GD20), as revealed by DNA microarray analysis. n=5.

**Group Probe set Gene title Fold change** (down)

***Growth factors*** 1389905_at EGF receptor x4

1391665_at FGF 7 x4

1368160_at IGF binding protein 1 x14

1368919_at Placental growth factor x9

1370941_at PDGF receptor, α polypeptide x9

1370887_at TGF β 1-induced transcript 1 x3

***Inflammation*** 1387969_at Chemokine (CXC motif) ligand 10 x4

1369633_at Chemokine (CXC motif) ligand 12 x5

1378015_at Chemokine (CXC motif) ligand 21β x4

1387868_at LPS binding protein x23

1372013_at Interferon induced transm. Prot. 1 x7

1387180_at Interleukin 1 receptor, type 2 x3

1369266_at Interleukin 13 receptor, α 2 x32

1388773_at TNF, α-induced protein 2 x5

1397221_at TNF receptor s/family, member 6 x2

***Matrix factors*** 1367594_at Biglycan x5

1370155_at Procollagen, type I, α 2 x8

1388116_at Collagen, type I, α1 x5

1370959_at Collagen, type III, α1 x5

1372439_at Procollagen, type IV, α 1 x4

1376099_at Collagen, type V, α1 x6

1370895_at Collagen, type V, α2 x9

1388142_at Chondroitin sulfate proteoglycan 2 x5

1370956_at Decorin x17

1393210_at Extracellular Matrix Protein 2 x12

1370234_at Fibronectin I x4

1374763_at Fibronectin Type III dom. Containing 1 x2

1382101_at Heparan Sulfate 2-O-sulfotransferase 1 x2

1394561_at Hyaluronan & Proteogly. Link Prot. 3 x2

1370462_at Hyaluronan mediated motility receptor x2

1376749_at Osteoglycin x27

1368655_at Proteoglycan Peptide Core Protein x4

***Neuronal factors*** 1393048_at Adrenergic Receptor, α 2a x2

1384667_at Galanin Receptor 2 x14

1389706_at GABA A Receptor x6

1367791_at Receptor (Calcitonin) Activity MP 1 x7

***Others*** 1367794_at Alpha-2-macroglobulin x155

1382751_at WAP four-disulfide Core Domain 10A x89

1368312_at Oxytocin x89

***Signaling*** 1372864_at Adenylate Cyclase 5 x5

1387253_at Guanylate Cyclase Activator 2b x72

1377993_at Guanine Nucleotide Binding Prot. 13, γ x6

1387809_at MAPKK 6 x2

1370445_at Phosphatidylserine-specific x13

Phospholipase A1

1371014_at Phospholipase C, β1 x3

1370585_at Protein Kinase C, β1 x4

***Vasculature*** 1384240_at Angiotensin II Receptor, Type I x5

1377783_at Angiopoietin 4 x3

1383641_at Endothelin Receptor Type A x5

1368463_at VEGF C x3

*WBC migration* 1374763_at Cerebral Endothelial CAM 1 x2

1389235_at ICAM 2 x3

1380236_at Integrin α 9 x4

1387144_at Integrin α 1 x3

1374933_at Melanoma CAM x3

1368474_at VCAM-1 x7
